# Inhibition of BET Proteins Reduces Right Ventricle Hypertrophy and Pulmonary Hypertension Resulting from Combined Hypoxia and Pulmonary Inflammation

**DOI:** 10.3390/ijms19082224

**Published:** 2018-07-30

**Authors:** Clovis Chabert, Saadi Khochbin, Sophie Rousseaux, Sylvie Veyrenc, Rebecca Furze, Nicholas Smithers, Rab K Prinjha, Uwe Schlattner, Christophe Pison, Hervé Dubouchaud

**Affiliations:** 1 Université Grenoble Alpes, Inserm U1055, Laboratoire de Bioénergétique Fondamentale et Appliquée, 38058 Grenoble, France; clovis.chabert@gmail.com (C.C.); uwe.schlattner@univ-grenoble-alpes.fr (U.S.); CPison@chu-grenoble.fr (C.P.); 2 CNRS UMR 5309, Inserm U1209, Université Grenoble Alpes, Institute for Advanced Biosciences, 38700 Grenoble, France; saadi.khochbin@univ-grenoble-alpes.fr (S.K.); sophie.rousseaux@univ-grenoble-alpes.fr (S.R.); 3 Université Grenoble Alpes, CNRS UMR 5553, Laboratoire d’Ecologie Alpine, 38058 Grenoble, France; sylvie.veyrenc@univ-grenoble-alpes.fr; 4 Epigenetics DPU, Immuno-Inflammation Therapy Area, GlaxoSmithKline R&D, Medicines Research Centre, Stevenage SG1 2NY, UK; rebecca.c.furze@gsk.com (R.F.); nick.n.smithers@gsk.com (N.S.); Rabinder.Prinjha@gsk.com (R.K.P.); 5 Centre Hospitalier Universitaire Grenoble Alpes, Université Grenoble Alpes, 38700 Grenoble, France

**Keywords:** Epigenetic, COPD, pulmonary hypertension, hypoxia, pulmonary inflammation

## Abstract

Pulmonary hypertension is a co-morbidity, which strongly participates in morbi-mortality in patients with chronic obstructive pulmonary disease (COPD). Recent findings showed that bromodomain-containing proteins, in charge of reading histone acetylation, could be involved in pulmonary arterial hypertension. Our aim was to study the effect of I-BET151, an inhibitor of bromodomain and extra-terminal domain (BET), on the right ventricle hypertrophy and pulmonary hypertension, induced by a combination of chronic hypoxia and pulmonary inflammation, as the two main stimuli encountered in COPD. Adult Wistar male rats, exposed to chronic hypoxia plus pulmonary inflammation (CHPI), showed a significant right ventricle hypertrophy (+57%, *p* < 0.001), an increase in systolic pressure (+46%, *p* < 0.001) and in contraction speed (+36%, *p* < 0.001), when compared to control animals. I-BET151 treated animals (CHPI-iB) showed restored hemodynamic parameters to levels similar to control animals, despite chronic hypoxia plus exposure to pulmonary inflammation. They displayed lower right ventricle hypertrophy and hematocrit compared to the CHPI group (respectively −16%, *p* < 0.001; and −9%, *p* < 0.05). Our descriptive study shows a valuable effect of the inhibition of bromodomain and extra-terminal domain proteins on hemodynamic parameters, despite the presence of chronic hypoxia and pulmonary inflammation. This suggests that such inhibition could be of potential interest for COPD patients with pulmonary hypertension. Further studies are needed to unravel the underlying mechanisms involved and the net benefits of inhibiting adaptations to chronic hypoxia.

## 1. Introduction

Pulmonary hypertension (PH) corresponds to an abnormal increase of the mean resting pulmonary arterial pressure to over 25 mmHg, which is associated with increased pulmonary vascular resistance, right ventricle (RV) hypertrophy and ultimately RV failure and death in those patients [1]. In chronic obstructive pulmonary disease (COPD), hypoxic PH (group 3 PH) occurrence is much more frequent compared with the healthy population, starting from 5% at the beginning (mild-to-moderate COPD) to more than 35% as the disease progresses over time (very severe COPD) [2]. PH is associated with a remodeling of small pulmonary arteries, which increases the hemodynamic resistances, leading to RV hypertrophy [1]. This could result in death as a consequence of right ventricle failure, making cardiovascular disorders the most frequent comorbidity in COPD [3]. This vascular remodeling of the pulmonary arteries is thought to result from chronic hypoxia (CH) exposure [2]. Indeed, CH induces cell proliferation by inhibiting anti-mitogenic factors, such as nitric oxide or prostacyclin, and by enhancing mitogenic stimuli, such as endothelin-1, platelet-derived growth factor or vascular endothelial-derived growth factor [4]. Moreover, CH could increase PH by the growth of extracellular matrix components [5]. In addition to CH, the chronic inflammation of COPD patients [6], enhanced by the oxidative stress and shear stress due to the friction of the red cells on the vessel walls, could contribute to the aberrant proliferation of vessel cells and to the development of cardiopulmonary pathologies [7].

Recent studies suggest that histone deacetylases (HDAC) could be involved in an epigenetic control of pulmonary arterial smooth muscle cells development and function, as shown in a newborn sheep model [8]. Targeting these epigenetic mechanisms with HDAC inhibitors successfully restored normal pulmonary arterial tension in models of pulmonary arterial hypertension (PAH) [9] and PH [10,11]. Besides PH related to group 3, PAH pathogenesis implicates several proteins whose expression is dependent on bromodomain (Brd)-containing proteins involved in the reading of acetylations [12,13]. Meloche et al. reported that treatment with JQ1, an inhibitor of Brd and extra-terminal domain (BET) proteins, could restore pulmonary arterial tension, as well as oncogene and cell cycle protein regulators, involved in the PAH control levels [13]. Indeed, in the hypoxia/Sugen 5416 (VEGF receptor antagonist) model [14], animals treated with such an inhibitor showed transcription levels of survival markers, NFATc2 and Bcl-2, similar to those of the control groups. JQ1 also strongly reduced the proliferation of pulmonary arterial smooth muscle cells, in parallel with an increase in apoptosis, which restored the cell cycle activity to that of control animals [13].

These results suggest that the effects of hypoxia on acetylation pathways [15] could have a specific role in these vascular alterations in COPD. However, inflammation is also known to affect acetylation signaling pathways [16] and could contribute, as another significant factor, to PH development. The specific role of these 2 stimuli on epigenetic factors has not been investigated in PH development COPD yet, although several tissues show modifications of acetylation signaling pathways in these patients [17,18].

We hypothesize that the use of inhibitors of BET proteins could be of potential interest for the control of the PH, which develops with the severity of the COPD, through epigenetic regulation over time. Our aim was to use I-BET151, an inhibitor of the BET domain, to determine if BET proteins are involved in the development of right ventricle hypertrophy and PH, induced on a model of CH combined with pulmonary inflammation (PI), two main stimuli encountered in COPD.

## 2. Results

### 2.1. Lipopolysaccharides (LPS) Instillations Are Associated with Signs of Lung Alterations

**Figure 1:**
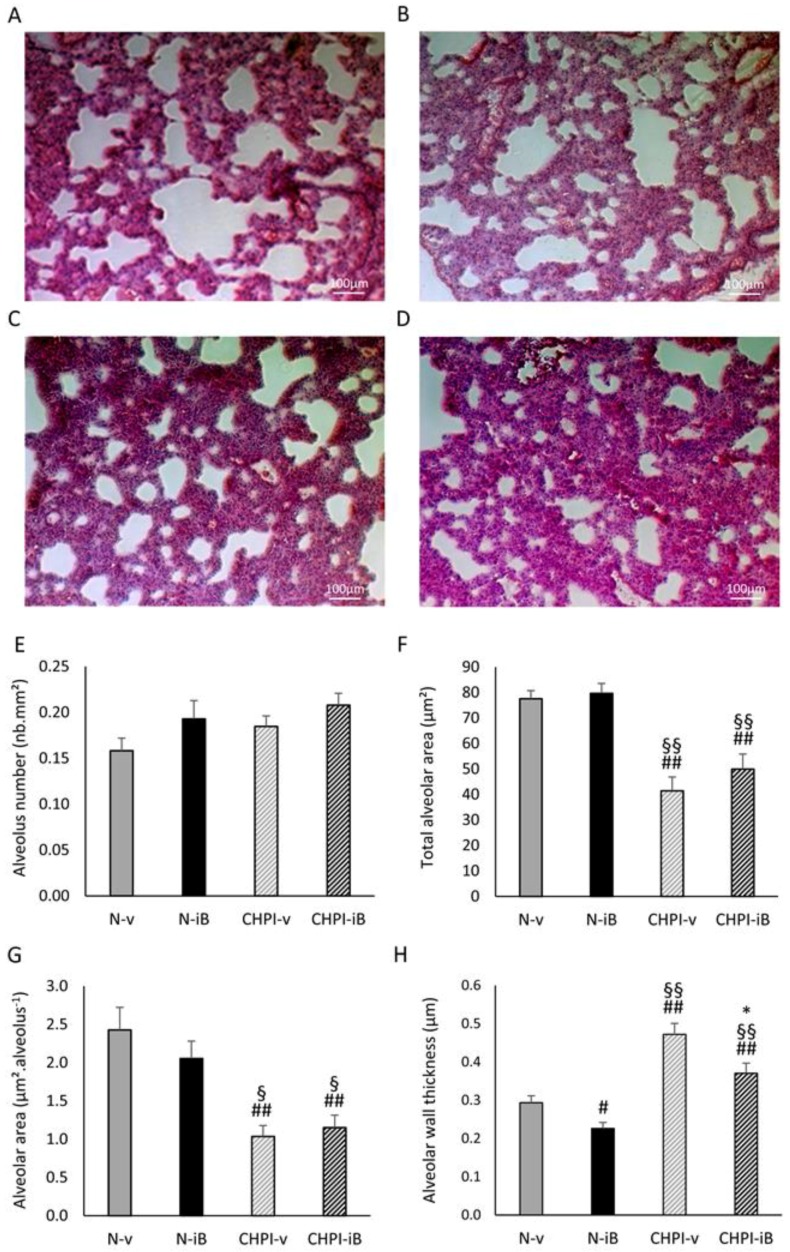
Effects of combined chronic hypoxia (CH) and pulmonary inflammation, induced by LPS instillations (PI), on the lung structure. Sectional view of lungs from N-v (**A**), N-iB (**B**), CHPI-v (**C**), and CHPI-iB (**D**) groups, under light microscope, after hematoxylin and eosin staining. Histograms represent the number of alveolus (**E**), the total alveolar area (**F**), the mean alveolus area (**G**), and the mean alveolar wall thickness (**H**) detected on each sectional view. All picture analyses were performed as described under the Materials and Methods section. N-v: Normoxia + vehicle; N-iB: Normoxia + I-BET151; CHPI-v: Chronic Hypoxia + Inflammation + vehicle; CHPI-iB: Chronic Hypoxia + Inflammation + I-BET151. (mean ± SEM; *n* = 7–8). #: diff. of N-v (#: *p* < 0.05; ##: *p* < 0.001); §: diff. of N-iB (§: *p* < 0.05; §§: *p* < 0.001); *: diff. of CHPI-v (*: *p* < 0.05).

Repeated LPS instillations had significant effects on lung structure, as depicted in Figure 1, including smaller alveolar size in the chronic hypoxia plus inflammation (CHPI) groups, whether there was I-BET151 administration (CHPI-iB, Figure 1D) or not (CHPI-v, Figure 1C), compared to the groups without LPS instillations (N-v and N-iB, Figure 1A,B, respectively). Image analysis showed that the alveolus number was similar in all groups (Figure 1E), but that total alveolar area was significantly smaller in groups with LPS instillations compared to non LPS groups (Figure 1F) as well as the alveolar area (Figure 1G). Moreover, the alveolar wall was significantly thicker in LPS-treated (CHPI-v and CHPI-iB) groups compared with non-LPS (N-v and n-iB) groups (Figure 1H). Additionally, the I-BET151 treatment did not have a significant effect on these LPS-induced alterations.

### 2.2. Inhibition of BET Proteins Reverses Hypertension of the Right Ventricle

**Figure 2:**
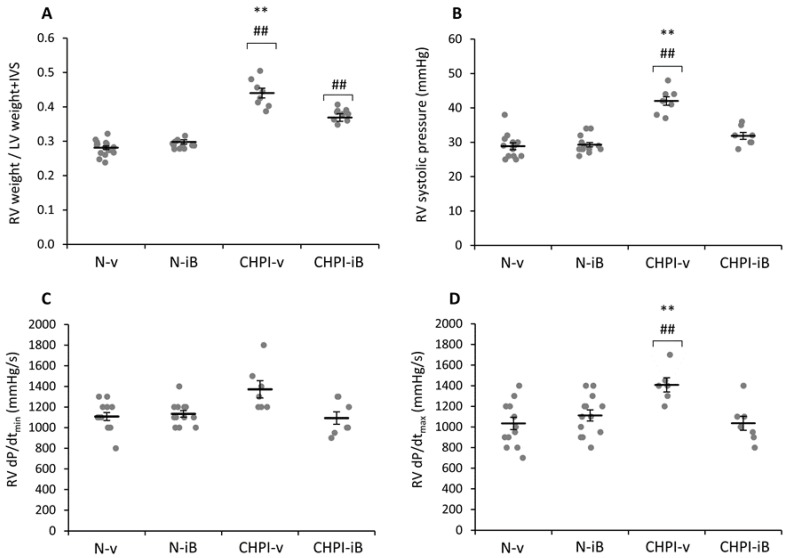
Effect of I-BET151 administration on the morphology and function of the right ventricle. Ratio of weights of the right ventricle to the left ventricle plus interventricular septum (**A**); right ventricular systolic pressure (**B**); right ventricular relaxation time; (**C**) and contraction time (**D**). IVS: Interventricular Septum; RV: Right Ventricle; N-v: Normoxia + vehicle; N-iB: Normoxia + I-BET (inhibitor of Bromodomains and Extra-Terminal domains); CHPI-v: Chronic Hypoxia + Inflammation + vehicle; CHPI-iB: Chronic Hypoxia + Inflammation + I-BET151. Horizontal bars represent the mean ± SEM; *n* = 7–16. ##: diff. from N-v and N-iB (*p* < 0.001); **: diff. from CHPI-v (*p* < 0.001).

After exposure to combined hypoxia and pulmonary inflammation, we found a significant RV hypertrophy, as judged by the higher RV/Left Ventricle + Interventricular Septum (LV + IVS) weight ratio (Fulton index; Figure 2A) in the Chronic Hypoxia plus Inflammation (CHPI-v) group compared to the Normoxia plus vehicle (N-v) group (+56%, *p* < 0.001). Treatment with I-BET151 did not change the Fulton index in the Normoxia group plus I-BET (N-iB) compared with the (N-v) group, but partially limited the RV hypertrophy under Hypoxia plus Inflammation (CHPI-iB group) to a level between CHPI-v and normoxic groups (N-v and N-iB; −16%, +32% and +23%, respectively, *p* < 0.001). RV pressures (Figure 2B) were significantly higher in the CHPI-v group compared to the control N-v group (+46%, *p* < 0.001). Treatment with I-BET151 did not change RV systolic pressure in normoxia (+2% of N-v, ns), but restored the values of the CHPI-iB group to levels similar to those of N-v and N-iB groups (respectively +10% and +9%, ns). No modifications were observed in the dP/dt_min_ parameter (Figure 2C), although the dP/dt_max_ was significantly higher in the CHPI-v group when compared to N-v, N-iB and CHPI-iB (+36%, +27% and +36%, respectively, *p* < 0.001). Thus, again, I-BET151 restored dP/dt_max_ values close to those of control groups (N-v and N-iB; +0.1% and −7%, respectively, ns).

### 2.3. Inhibition of BET Proteins Has No Effect on Left Ventricle

**Figure 3:**
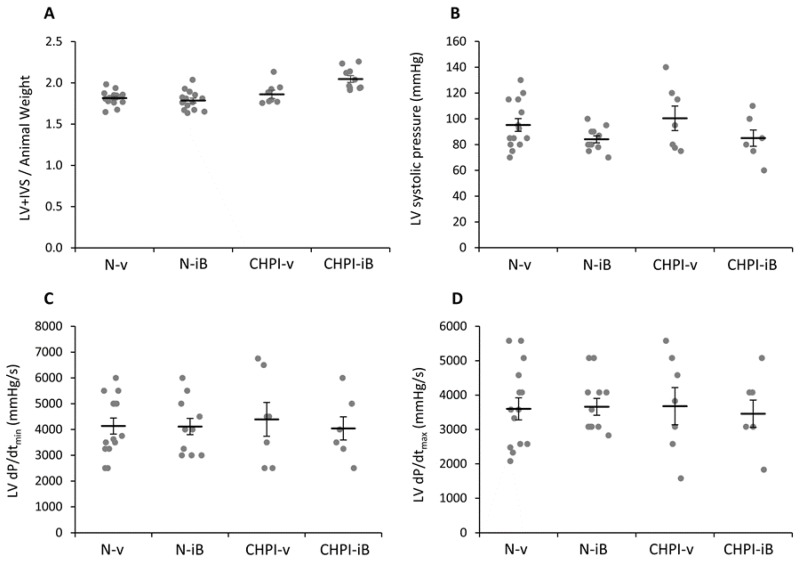
Effect of I-BET151 administration on the morphology and function of the left ventricle. Weight of left ventricle + interventricular septum normalized to the animal body weight (**A**); Left ventricular systolic pressure (**B**); Left ventricular relaxation time; (**C**) and contraction time (**D**). LV: Left Ventricle; IVS: Interventricular Septum; I-BET: Inhibitor of Bromodomains and Extra-Terminal domains; N-v: Normoxia + vehicle; N-iB: Normoxia + I-BET151; CHPI-v: Chronic Hypoxia + Inflammation + vehicle; CHPI-iB: Chronic Hypoxia + Inflammation + I-BET151. Horizontal bars represent the mean ± SEM; *n* = 6–16.

In our model, the LV + IVS weight, expressed as a function of the body weight, was not altered by CH or PI (Figure 3A). Likewise, analysis of the LV systolic pressure, presented in Figure 3B, did not show any significant differences in N-iB, CHPI-v or CHPI-iB when compared to the N-v group (respectively −12%, +5%, −11%, ns). Contraction and relaxation speed parameters (Figure 3C,D) were neither modified by CH and PI, nor by I-BET151 administration, when compared to N-v animals.

### 2.4. Inhibition of BET Proteins Blunts Hematocrit Increase Induced by Chronic Hypoxia and Inflammation

**Figure 4:**
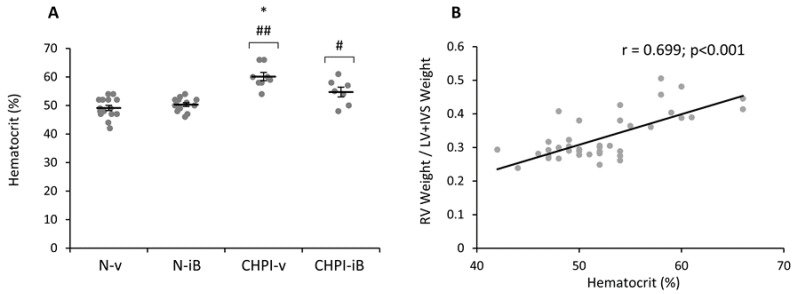
Hematocrit levels of animals in each groups (**A**); Relationship between right ventricle hypertrophy and the hematocrit level of the animal treated with vehicle or with I-BET151 (**B**). IVS: Interventricular Septum; RVDP: Right Ventricle Diastolic Pressure; N-v: Normoxia + vehicle; N-iB: Normoxia + I-BET151; CHPI-v: Chronic Hypoxia + Inflammation + vehicle; CHPI-iB: Chronic Hypoxia + Inflammation + I-BET151. Horizontal bars represent the mean ± SEM; *n* = 7–15. #: diff. of N-v and N-iB (#: *p* < 0.05; ##: *p* < 0.001); *: diff. of CHPI-v (*p* < 0.05).

The hematocrit level presented in Figure 4A was higher in CHPI-v group when compared to N-v (+22%, *p* < 0.001). However, while no difference was observed between N-v and N-iB (+2.4%, ns), the hematocrit value in CHPI-iB was significantly lower than in CHPI-v (-9%, *p* < 0.05), although still higher than in the N-v and N-iB groups (respectively +11%, +9%, *p* < 0.05). Furthermore, there was a significant correlation between the hematocrit and Fulton index in both groups, with or without the I-BET151 treatment (r = 0.699, *p* < 0.001; Figure 4B).

## 3. Discussion

We report clear evidence that the inhibition of BET proteins is able to restore the right ventricle hemodynamic parameters of animals subjected to combined chronic hypoxia (CH) and pulmonary inflammation (PI), and partially correct the resulting RV hypertrophy.

Pulmonary hypertension (PH) can be induced in several ways in animal models, including the pulmonary arterial banding [19], monocrotaline injections, hypoxia/Sugen exposure [13], and CH exposure [20], resulting in increases in the pulmonary arterial tension from 30% to over 150% of the control values. The increase from 28 mmHg to 42 mmHg in systolic pressure, which we observed in our model, is in accordance with those observed in other models and corresponds to a mild PH.

However, all of these models are not equivalent. As an example, pulmonary arterial banding induces a mechanical increase of the vascular resistance, which restrains drug testing possibilities. The monocrotaline models can be relevant to mimic idiopathic PAH [21], but the confusing restoration of PAH by drugs, well known to induce idiopathic PAH in humans, decreases the relevance of this model for drug testing [22]. The hypoxia/Sugen rodent model is a very interesting model for idiopathic PAH, but not for hypoxic PH, because damages continue to develop even after the return to normoxia [21]. To mimic the alterations encountered with COPD PH, the CH exposure of animals could, at first sight, be a relevant model, but the lack of PI, which plays a key role in the development of COPD PH [2], is a substantial default. For all these reasons, we proposed to combine a CH and PI stimulus to obtain a cellular micro-environment of the pulmonary vessels, as close as possible to that observed in COPD patients. Indeed, CH is well known to induce pulmonary vasoconstriction, medial hypertrophy and an increased muscularization of the small arteries with elevated smooth muscle α-actin [23]. Morell et al. [24] showed a significant increase in the PH, Fulton index, hematocrit, and wall thickness of muscle arteries after 8 days of hypoxia exposure (FiO_2_: 10%). Repeated instillations of lipopolysaccharides have been reported to induce lung remodeling (hyperinflation, hypertrophy, and an alveolar enlargement) associated with a reduced VEGF expression and an increase in the tissue inhibitor of matrix metalloproteinases-1 (TIMP1) protein, two proteins linked to vascular remodeling [25]. This remodeling is accompanied by a chronic inflammatory response of lungs, which could make it a suitable model of COPD [26]. The use of LPS instillations in our model induced significant modifications of the lung structure, with a smaller alveolar area and thicker alveolar wall, as can be judged from the analysis of lung sections after hematoxylin and eosin staining, independently of the I-BET151 administration. We did not observe the same alveolar size as others [26] but this is probably due to the lung tissue collection process. Indeed, in most studies, lung tissues are collected after the inflation of fixative under mild pressure. Due to our protocol, we were unable to do so and have simply frozen a portion of lung without any further treatment. Interestingly, previous work performed in our laboratory shows that the combination of PI and PH induces alterations of locomotor muscle epigenetic landscape, similar of those observed in COPD patients [27]. The significant differences observed in our studies, along with data from literature on the same model, suggest that there is pulmonary inflammation, thus making this methodological approach a good model to study the consequences of such pulmonary inflammation. Thus, combining two weeks of PI, followed by one week of PI plus CH before beginning the I-BET151 treatment, appears to be a relevant way to replicate the COPD pulmonary hypertension.

Brd-containing proteins and extra-terminal domains (BET) proteins were recently shown to be involved in the development of PAH in humans and animal models of PAH [13]. These results, together with the restoration of the pulmonary arterial tension to levels, similar to controls by the inhibition of BET proteins in a hypoxia/Sugen model [13], open new perspectives for this drug family for the treatment of PAH. However, the effects observed by Meloche et al. [13] are only related to the hypoxia condition. Indeed, a model associating a hypoxic condition with an inflammatory state, such as the one presented here, could be more relevant as these are the two main stimuli encountered in COPD PH.

In our study, measurements of the LV did not show any effect of the I-BET151 in hemodynamic nor morphological parameters. However, I-BET151 treatment appeared to be able to totally restore the RV systolic pressure to a value similar to those in the control group. Moreover, the partial hypertrophy of the RV, observed in the group treated with I-BET151 and subjected to CH and PI, strongly suggests that the drug could restore the hemodynamic parameters of the pulmonary circulation first, since the RV systolic pressure under I-BET151 did not differ to that of controls. This result is in accordance with those reported by Meloche et al., showing that BET inhibition restores the survival/proliferation of pulmonary arterial smooth muscle cells [13]. These effects rely specifically on the involvement of the targets of I-BET151 and are unlikely due to changes in the level of lung alterations induced by LPS administration, since similar changes were obtained both in I-BET151 and vehicle groups. In this regard, targeting the RV hypertrophy without a change in PH worsened the RV dysfunction in a model of pulmonary artery banding in rats treated with a HDAC inhibitor [19]. These authors report a severe impairment of the RV function associated with apoptosis, decreased angiogenesis, and excessive fibrosis. The partial restoration of hematocrit and RV hypertrophy that we observed with I-BET151, probably due to the one-week of CH exposure before starting I-BET administration, are supplemental clues that I-BET151 is a valuable drug to reverse the PH, which involves an altered acetylation profile of the artery smooth muscle cells [12].

Brd-containing proteins are known to be ubiquitous in mammals and to be involved in pathways controlling the acclimation to various conditions, such as CH. Therein, the partial decrease in the hematocrit level, measured in animals treated with I-BET151 under CH and PI, seems to indicate that this drug also inhibits the polycythemia, classically shown after CH exposure [28]. CH induces a major cellular stress, including disturbances in energy homeostasis and some of the mechanisms involved in the acclimation to CH that imply acetylation pathways [15]. For example, acetylation of the Hypoxia Inducible Factor-2α (HIF-2α) is required to initiate polycythemia, induced by an increase in erythropoietin (Epo) [29]. Furthermore, the expression of HIF-2α target genes is increased by hyper-acetylation of histones, H3 and H4, in the proximal promoter/enhancer region of HIF-2α during hypoxia exposure [30]. The decrease of the polycythemia could be the result of an inhibition of the acetylation pathways by the I-BET151 that could then lead to an impairment of the HIF-2α pathway. Furthermore, the partial restoration of the hematocrit could be explained by the kinetics of our model that exposes animals to CH during one week before treatment with I-BET151 until sacrifice. Thereby, the organisms had already initiated the acclimation to CH [24] and PI [25] prior to treatment, possibly explaining the partial increase in the hematocrit and RV hypertrophy, seen in CHPI-iB group.

A high hematocrit is associated with a lower pulmonary microcirculation, leading to a decrease in gas exchanges that would impair blood oxygenation [31]. Repeated hemodilutions improve pulmonary gas exchange, central hemodynamics, and exercise tolerance in patients with severe COPD and PH [32]. The decrease of hematocrit by I-BET151 treatment could be a supplemental asset to the reduction of blood viscosity and the restoration of the hemodynamics parameters in COPD with PH.

## 4. Material and Methods

### 4.1. Animals

**Figure 5:**
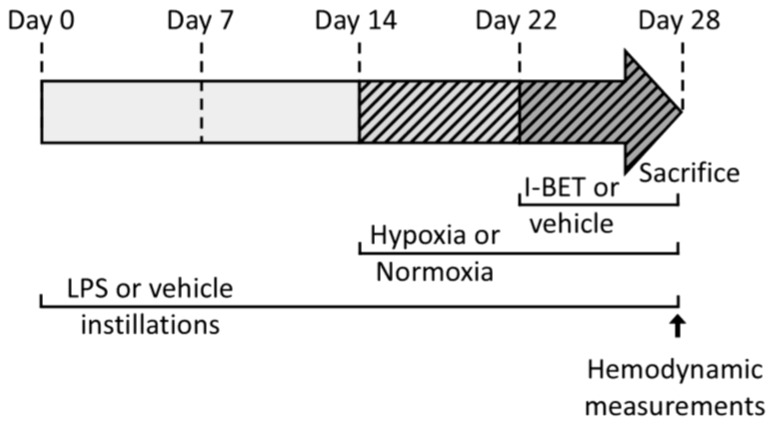
Chronogram of experimental protocol. I-BET: Inhibitor of Bromodomains and Extra-Terminal domains; LPS: Lipopolysaccharides. LPS or its vehicle instillations were performed for 28 days. For the last 14 days, animals were exposed to either a normoxic or normobaric hypoxic (FiO_2_ 10%) environment. For the last 7 days, animals were treated with I-BET151, or its vehicle, until the time of sacrifice (Day 28), as previously described [27].

Male adult Wistar rats (4 month-old, 392 ± 7 g) were used in this study. Procedures were carried out in accordance with European Directives 86/609/EEC, 2010/63/UE and the GSK policy on the care, wellbeing and treatment of animals. All the procedures were approved by the local ethics committee, affiliated with the animal facility of the university (D3842110001), on 21/01/2013, and agreed to by the French Ministry of Research (345_LBFA-U1055). Animals were assigned to one of the four following groups (Figure 5): Normoxia + vehicle (N-v), normoxia + I-BET151 (N-iB), chronic hypoxia + pulmonary inflammation + vehicle (CHPI-v), chronic hypoxia + pulmonary inflammation + I-BET151 (CHPI-iB).

At sacrifice, hematocrit measurements were performed using glass capillaries and centrifuged at 17 000 g for 3 min (Haemofuge A, Heraus, Grésy-sur-Aix, France). The RV and the left ventricle + Interventricular Septum (LV + IVS) were dissected, separated and weighted. RV hypertrophy was assessed, post mortem, as the weight ratio of the right ventricle free wall to the LV + IVS, known as the Fulton index. Lung portions were collected and frozen in liquid nitrogen for subsequent morphological analysis.

### 4.2. Right Ventricle Hypertrophy Induction by Pulmonary Inflammation (PI) and Chronic Hypoxia (CH)

PI was induced by the bi-weekly intratracheal administration of lipopolysaccharides (LPS) 0.4 mg/mL, diluted in NaCl 0.9% for 4 weeks (1 mL/kg of body weight; *E. coli*, serotype O55:B5; Sigma-Aldrich Chemical Co.™, St Louis, MO, USA). This model is reported to induce airway remodeling, with similarities to those observed in COPD patients [25,26].

During the last 14 days of the PI protocol, animals were exposed to a either chronic normoxia (N groups) or normobaric hypoxia (CH groups, FiO_2_: 10%). The hypoxia chamber was opened for 45–60 min per day to weigh animals and perform LPS administration and nursing, as previously described [27].

### 4.3. Bromodomain and Extra-C-Terminal Domain Inhibitor (I-BET151) Administration

The I-BET151, also referenced as GSK1210151 (GlaxoSmithKline™, London, UK), was administered daily by oral gavage (10 mg/mL/kg of body weight in methyl cellulose 400 cp, 1% in H_2_O) during the last week before sacrifice. Untreated animals received the same volume of vehicle.

### 4.4. Lung Morphometry Analysis

Sections of frozen lung were cut on glass slides, air dried, and then stained using hematoxylin and eosin. The slides were immersed in Mayer’s hematoxylin (DAKO, Glostrup, Denmark) for 30 s, rinsed with tap water until clear, dipped in eosin (Sigma-Aldrich Chemical Co.™, St Louis, MO, USA) for 10 s, and again rinsed with tap water. The slides were air-dried at room temperature and then dipped twice in 95% ethanol, twice in 100% ethanol, twice in 50% ethanol, 50% xylene solution, and twice in 100% xylene. Finally, coverslips were mounted using a Permount^®^ medium (Fisher Scientific, Waltham, MA, USA). Slides were then observed under a standard light microscope, and images were analyzed for structural indices, including alveolar density and size, and epithelial wall thickness using ImageJ software.

### 4.5. Hemodynamic Measurements

Heart rate, systolic pressure in the Right Ventricle (RV) and Left Ventricle (LV) were measured, in situ at sacrifice (Figure 5), using a Millar microprobe (SPR-249A, Millar Inc., Gulf Fwy, Houston, TX, USA), associated with a transducer (TC-100, Millar Inc.) and an electromanometer (Gould 2200 recorder, Millar Inc.). Animals were anesthetized with sodium pentobarbital (50 mg/kg of body weight). The pressure catheter was introduced in the jugular vein up to the RV or in the carotid artery up to the LV, as described previously [13,33]. Relaxation and contraction times were obtained by deriving the curve of the ventricle pressures (respectively dP/dt_min_ and dP/dt_max_).

### 4.6. Statistical Analysis

All the data are presented as mean ± SEM and were analyzed using a one-way Analysis of Variance (ANOVA) as they were normally distributed. If an effect was detected, a Holm-Sidak post-hoc analysis was performed. Correlation coefficients were calculated as a Pearson product moment. Statistical significance was accepted at *p* < 0.05.

## 5. Conclusions

Our study describes novel beneficial effects of I-BET151 under conditions of pulmonary hypertension, induced by chronic hypoxia and pulmonary inflammation. This novel insight argues for a translation of I-BET drugs from animal models to patients, in order to reverse group 3 PH in COPD, for which no registered treatments exist [1,34]. Indeed, I-BET151 treatment could be an asset to the restoration of hemodynamic parameters, but further experiments are mandatory to evaluate the net balance of benefits to risks before translation to patients with PH secondary to COPD.
